# Hormone replacement therapy decreases the risk of tinnitus in menopausal women: a nationwide study

**DOI:** 10.18632/oncotarget.24452

**Published:** 2018-02-08

**Authors:** Hsin-Chien Chen, Chi-Hsiang Chung, Vincent C.F. Chen, Yu-Chi Wang, Wu-Chien Chien

**Affiliations:** ^1^ Department of Otolaryngology-Head and Neck Surgery, Tri-Service General Hospital, National Defense Medical Center, Taipei, Taiwan; ^2^ Department of Medical Research, Tri-Service General Hospital, National Defense Medical Center, Taipei, Taiwan; ^3^ School of Public Health, National Defense Medical Center, Taipei, Taiwan; ^4^ Engineering Science, Loyola University Chicago, Chicago, IL, USA; ^5^ Department of Obstetrics and Gynecology, Tri-Service General Hospital, National Defense Medical Center, Taipei, Taiwan

**Keywords:** hormone replacement therapy (HRT), tinnitus, menopausal syndrome

## Abstract

The incidence and risk of tinnitus associated with hormone replacement therapy (HRT) in menopausal women have not yet been fully examined. We examined the medical records of menopausal women aged between 45 and 79 years from Taiwan's National Health Insurance Research Database of records between 1 January 2000 and 31 December 2010 to establish matched cohorts (13,920 HRT users and 41,760 nonusers). The incidence of tinnitus in HRT users and nonusers were matched 1:3 based on propensity-score matching over this ten year period. The Cox regression hazard model was used to identify risk factors of tinnitus, and results indicate that a significantly lower percentage of HRT users (*P* = 0.017) developed tinnitus in comparison with nonusers (0.43%, 60/13, 920 vs. 0.59%, 246/41, 760). Using Cox regressions analysis after adjustments for age and other variables (adjusted hazard ratio: 0.505 (95% confidence interval, 0.342–0.756)), we were also able to show that HRT users appeared to have a reduced risk of developing tinnitus in comparison with nonusers. Based on our observation of the lower incidence of tinnitus among HRT users in this cohort, we speculate that HRT may have provided potential benefits on the management and prevention of tinnitus among menopausal women.

## INTRODUCTION

Menopausal syndrome refers to symptoms such as hot flashes, mood changes, fatigue, stress, tiredness, and vaginal dryness and itching that occur when women reach to the age that their ovaries can no longer function well enough to provide hormones such as estrogen and progesterone. Hormone replacement therapy (HRT) has been widely used to treat these symptoms; but despite the wide use, some studies have indicated that HRT is associated with a risk of coronary heart disease, stroke, breast cancer and endometrial cancer [1–4] while some articles have suggested that HRT may be beneficial to prevent osteoporosis and dementia in postmenopausal women [3, 5].

Tinnitus is an annoying symptom that involves a complex pathophysiology of peripheral and central auditory pathways that becomes even more complicated with psychological events [6]. It has been associated with sudden hearing loss, noise trauma, presbyacusis, administration of ototoxic drugs, microvascular compression of auditory nerves, vestibular schwannoma, temporomandibular joint disorders, and even mood, depression and stress [6]. There is a 10–15% prevalence rate and the symptom impairs quality of life in 1–2% of the population [6]. A recent study indicated that tinnitus may be encountered by women during their menopausal or immediate postmenopausal period and found HRT to be helpful for the treatment [7]. This is consistent with studies that suggested reproductive hormones to be possible factors that contribute to tinnitus development [8, 9].

As the relationship between tinnitus and menopause has rarely been reported and that no large survey has been conducted to investigate associations between tinnitus and HRT for menopausal women. We assessed the incidence of tinnitus and the effect of HRT on subsequent risk of tinnitus in menopausal women and conducted a nationwide population-based cohort study by analyzing data from a nationwide medical database (the National Health Insurance Research Database).

## RESULTS

### Characteristics of prevalence of tinnitus, covariates and comorbidities at the end of follow-up for HRT users compared with nonusers in menopausal women

**Figure 1:**
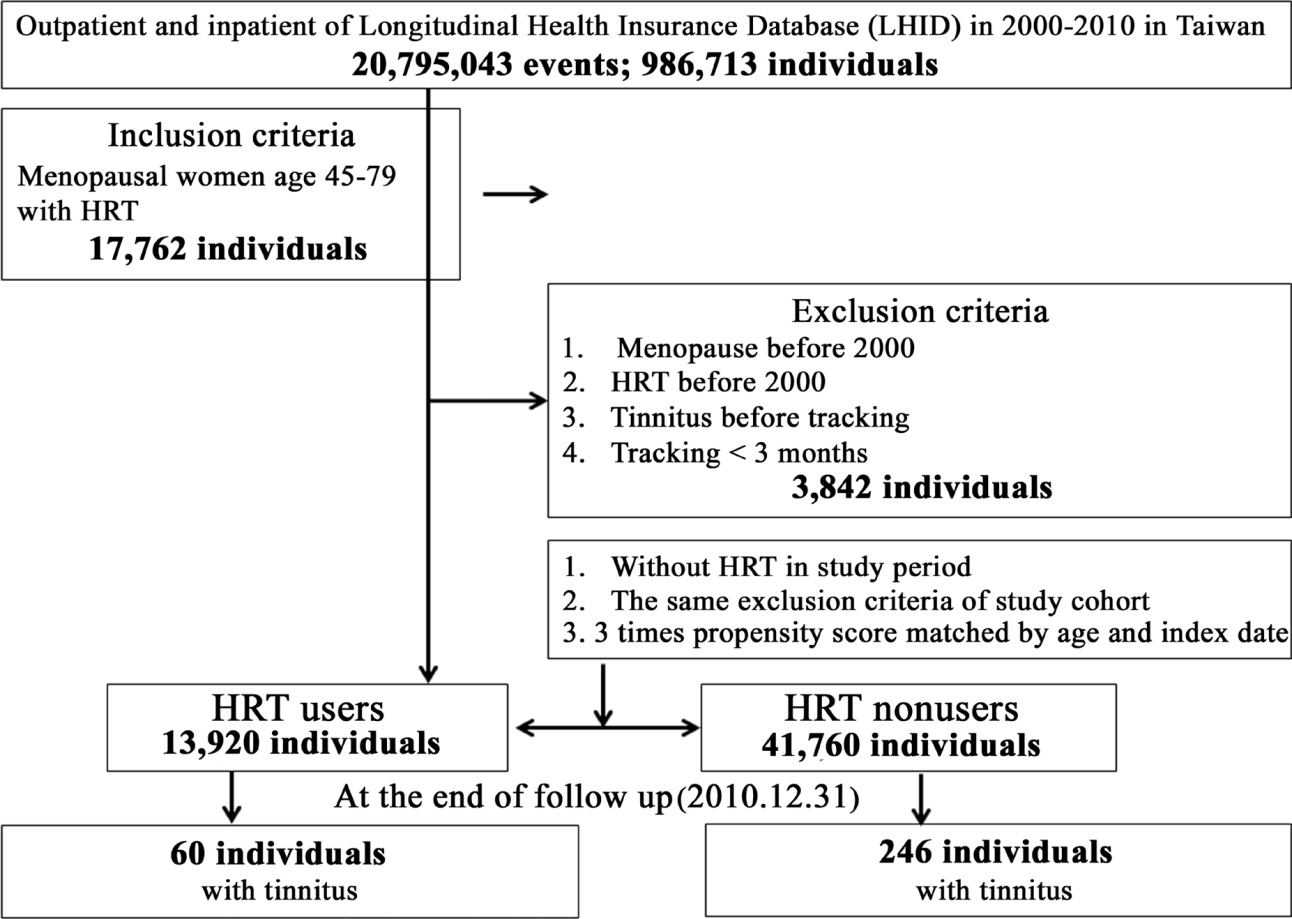
Flowchart of study sample selection from the National Health Insurance Research Database in Taiwan

According to the included data from January 1, 2000 to December 31, 2010, a total number of 13,920 HRT users were eligible and a matched 41,760 nonusers were selected as members of the control group (Figure 1). The baseline of the matched-cohort study indicated no significant difference between HRT users and nonusers with regard to age distribution. At the end of the follow-up (Supplementary Table 1), 60 HRT users (0.43%, 60/13,920) and 246 nonusers (0.59%, 246/41,760) developed tinnitus, indicating a lower prevalence of tinnitus in HRT users with a statistical significance of *P* = 0.017. The average of the follow-up period was 6.20 ± 5.26 yrs.

Regarding other variables, there was a significantly higher percentage of HRT users who have catastrophic illness (41.47% vs. 32.96%; *p <* 0.001), depression (17.41% vs. 9.46%; *p <* 0.001), insomnia (3.10% vs. 1.78%; *p <* 0.001), osteoporosis (3.19% vs. 2.82%; *P* = 0.012), hyperlipidaemia (5.15% vs. 4.16%; *p <* 0.001), and systemic lupus erythematosus (SLE) (1.12% vs. 0.63%; *p <* 0.001). HRT users paid significantly higher premiums when compared to nonusers (NT$) ≥35,000 (6.72% vs. 0.17%; *p <* 0.001) and lived in areas that were significantly different from nonusers (urbanization level 1 (27.76% vs. 27.16%), 3 (12.51% vs. 7.35%), and 4 (22.84% vs. 17.84%)). More HRT users receive their prescription from medical centers (34.08% vs. 28.25%) and local hospitals (29.91% vs. 24.63%) when compared to nonusers (*p <* 0.001).

### Kaplan–Meier model for cumulative risk of tinnitus in menopausal women with or without HRT

**Figure 2:**
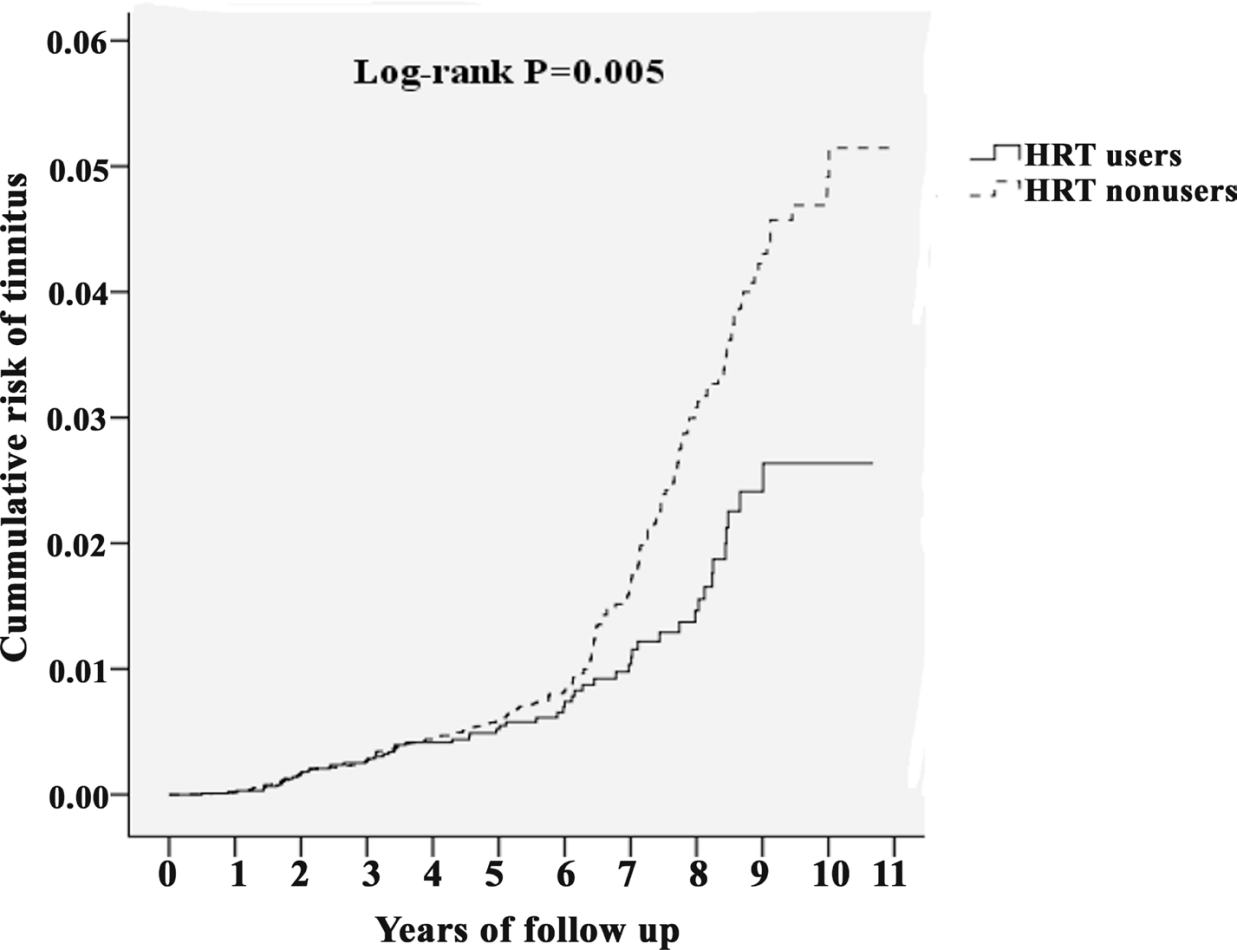
Kaplan–Meier curves for the cumulative risk of tinnitus among age 45–79 menopausal women with or without HRT using the log-rank test

The cumulative incidence curve of tinnitus for the HRT users cohort was significantly lower than the comparison cohort after age and variable adjustments (Figure 2, Log-rank test *P* = 0.005). In menopausal women with HRT, risk of tinnitus decreased progressively with increasing duration of follow-up.

### Hazard ratio (HR) and incidence of tinnitus stratified by age and variables by using Cox regression for HRT users vs. nonusers

A decreased risk of developing tinnitus, with an adjusted HR of 0.505 (95% CI, 0.342–0.756), was observed in HRT users using Cox regressions analysis after adjustments for age and variables (Table 1). The individuals with catastrophic illness, diabetes mellitus (DM), stroke, and ischemic heart disease (IHD) had a significantly lower risk of developing tinnitus. Higher income, hypertension (HT) and urban living was associated with significantly higher risk of developing tinnitus.

**Table 1:** Factors for tinnitus at the end of follow-up using Cox regression

Variables	Crude HR	95% CI	95% CI	*P*	Adjusted HR	95% CI	95% CI	*P*
**HRT**								
Without	Reference				Reference			
With	0.717	0.540	0.950	0.021	0.505	0.342	0.756	0.001
**Catastrophic illness**								
Without	Reference				Reference			
With	0.453	0.341	0.600	<0.001	0.544	0.412	0.768	<0.001
**Insured premium (NT$)**								
<18,000	Reference				Reference			
18,000–34,999	0.990	0.652	1.504	0.962	1.319	0.705	2.296	0.333
≥35,000	4.703	3.094	7.149	<0.001	5.524	3.141	8.670	<0.001
**Otitis media**								
Without	Reference				Reference			
With	0.000	-	-	0.598	0.000	-	-	0.606
**Meniere's disease**								
Without	Reference				Reference			
With	0.000	-	-	0.442	0.000	-	-	0.465
**Hearing loss**								
Without	Reference				Reference			
With	5.796	0.025	19.572	0.987	5.664	0.011	18.595	0.981
**DM**								
Without	Reference				Reference			
With	0.575	0.437	0.756	<0.001	0.496	0.363	0.658	<0.001
**HT**								
Without	Reference				Reference			
With	1.728	1.379	2.617	<0.001	2.311	1.804	2.986	<0.001
**Depression**								
Without	Reference				Reference			
With	1.216	0.875	1.690	0.244	1.235	0.804	1.788	0.158
**Insomnia**								
Without	Reference				Reference			
With	0.000	-	-	0.088	0.000	-	-	0.942
**Stroke**								
Without	Reference				Reference			
With	0.542	0.322	0.910	0.021	0.522	0.307	0.888	0.013
**Dementia**								
Without	Reference				Reference			
With	0.000	-	-	0.195	0.000	-	-	0.972
**CKD**								
Without	Reference				Reference			
With	0.295	0.094	0.919	0.035	3.211	0.386	30.295	0.397
**Osteoporosis**								
Without	Reference				Reference			
With	0.000	-	-	0.454	0.000	-	-	0.975
**Nephritis/Nephrotic syndrome/Nephrosis**								
Without	Reference				Reference			
With	0.207	0.077	0.556	0.002	0.198	0.022	1.298	0.098
**Hyperlipidaemia**								
Without	Reference				Reference			
With	1.922	1.319	2.802	0.001	1.464	0.897	2.025	0.194
**SLE**								
Without	Reference				Reference			
With	0.000	-	-	0.541	0.000	-	-	0.918
**IHD**								
Without	Reference				Reference			
With	0.534	0.330	0.840	0.007	0.572	0.335	0.911	0.018
**DVT**								
Without	Reference				Reference			
With	0.000	-	-	0.595	0.000	-	-	0.787
**Urbanization level**								
1 (The highest)	1.748	1.015	2.515	0.043	1.830	1.233	2.719	0.004
2	1.588	0.926	2.238	0.157	1.472	1.029	2.164	0.038
3	1.333	0.913	2.188	0.255	1.181	0.729	1.988	0.517
4 (The lowest)	Reference				Reference			
**Level of care**								
Hospital center	0.769	0.562	1.053	0.101	0.622	0.459	1.013	0.062
Regional hospital	0.895	0.867	1.165	0.410	0.817	0.612	1.101	0.164
Local hospital	Reference				Reference			

Abbreviations: HR = hazard ratio, CI = confidence interval, Adjusted HR: Adjusted variables listed in the table.

We further investigated factors associated with tinnitus by applying Cox regression analysis to the stratified variables in Table 2 of HRT users and nonusers. A significantly lower adjusted HR was observed among HRT users (catastrophic illness HR: 0.634 (*p <* 0.001); no catastrophic illness HR: 0.507 (*P* = 0.030)), which was independent of whether or not the individual had a catastrophic illness. A significantly lower adjusted HR (Table 2) among HRT users was observed among individuals with or without comorbidities that include DM and HTN. As no otologic disease was found among the HRT users in the Database, its interference has been excluded in this study. There was also a significantly lower adjusted HR (Table 2) among HRT users who did not have comorbidities.

**Table 2:** Factors for tinnitus stratified by variables assessed through Cox regression analysis

HRT (With vs. without)	With			Without			Ratio	Adjusted HR	95% CI	95% CI	*P*
Stratified	Event	PYs	Rate (per 10^5^ PYs)	Event	PYs	Rate (per 10^5^ PYs)					
**Total**	60	84, 573.38	70.94	246	260, 443.28	94.45	0.751	0.505	0.342	0.756	0.001
**Catastrophic illness**											
Without	48	45, 098.53	106.43	198	178, 999.89	110.61	0.962	0.634	0.418	0.963	<0.001
With	12	39, 474.85	30.40	48	81, 443.39	58.94	0.516	0.507	0.014	0.812	0.030
**Insured premium (NT$)**											
<18,000	12	51, 762.97	23.18	246	258, 653.66	95.11	0.244	0.271	0.144	0.496	<0.001
18,000-34,999	26	27, 571.22	94.30	0	1, 159.61	0.00	-	-	-	-	-
≥35,000	22	5, 239.19	419.91	0	630.01	0.00	-	-	-	-	-
**Otitis media**											
Without	60	84, 265.23	71.20	246	259, 298.32	94.87	0.751	0.505	0.342	0.756	0.001
With	0	308.15	0.00	0	1, 144.96	0.00	-	-	-	-	-
**Meniere's disease**											
Without	60	84, 573.38	70.94	246	260, 213.41	94.54	0.750	0.504	0.345	0.759	0.001
With	0	0.00	#DIV/0!	0	229.87	0.00	-	-	-	-	-
**Hearing loss**											
Without	58	82, 689.18	70.14	246	254, 847.14	96.53	0.727	0.513	0.299	0.678	<0.001
With	2	1, 884.20	106.15	0	5, 596.14	0.00	-	-	-	-	-
**DM**											
Without	50	56, 529.05	88.45	205	180, 659.64	113.47	0.779	0.595	0.314	0.777	0.001
With	10	28, 044.33	35.66	41	79, 783.64	51.39	0.694	0.486	0.112	0.845	0.019
**HT**											
Without	36	60, 958.83	59.06	136	178, 057.38	76.38	0.773	0.551	0.328	0.922	0.021
With	24	23, 614.55	101.63	110	82, 385.90	133.52	0.761	0.441	0.205	0.946	0.034
**Depression**											
Without	60	70, 394.59	85.23	205	236, 810.29	86.57	0.985	0.604	0.443	0.975	0.018
With	0	14, 178.79	0.00	41	23, 632.99	173.49	0.000	0.000	-	-	0.915
**Insomnia**											
Without	60	81, 937.02	73.23	246	255, 828.31	96.16	0.762	0.505	0.342	0.756	0.001
With	0	2, 636.36	0.00	0	4, 614.97	0.00	-	-	-	-	-
**Stroke**											
Without	60	80, 051.65	74.95	231	235, 283.92	98.18	0.763	0.528	0.337	0.794	0.001
With	0	4, 521.73	0.00	15	25, 159.36	59.62	0.000	0.000	-	-	0.945
**Dementia**											
Without	60	83, 417.27	71.93	246	255, 410.31	96.32	0.747	0.505	0.342	0.756	0.001
With	0	1, 156.11	0.00	0	5, 032.97	0.00	-	-	-	-	-
**CKD**											
Without	60	82, 623.49	72.62	243	251, 308.70	96.69	0.751	0.505	0.342	0.756	0.001
With	0	1, 949.89	0.00	3	9, 134.58	32.84	0.000	0.000	-	-	0.998
**Osteoporosis**											
Without	60	82, 192.67	73.00	246	253, 508.46	97.04	0.752	0.505	0.342	0.756	0.001
With	0	2, 380.71	0.00	0	6, 934.82	0.00	-	-	-	-	-
**Nephritis/Nephrotic syndrome/Nephrosis**											
Without	60	80, 017.18	74.98	245	243, 651.51	100.55	0.746	0.513	0.349	0.768	<0.001
With	0	4, 556.20	0.00	1	16, 791.77	5.96	0.000	0.000	-	-	0.999
**Hyperlipidaemia**											
Without	48	78, 979.62	60.78	228	247, 446.26	92.14	0.660	0.539	0.311	0.897	0.010
With	12	5, 593.76	214.52	18	12, 997.02	138.49	1.549	0.351	0.284	4.295	0.764
SLE											
Without	60	83, 717.09	71.67	246	258, 658.68	95.11	0.754	0.505	0.342	0.756	0.001
With	0	856.29	0.00	0	1, 784.60	0.00	-	-	-	-	
**IHD**											
Without	60	75, 596.82	79.37	226	229, 057.26	98.67	0.804	0.533	0.384	0.855	0.001
With	0	8, 976.56	0.00	20	31, 386.02	63.72	0.000	0.000	-	-	0.986
**DVT**											
Without	60	84, 098.55	71.34	246	259, 473.51	94.81	0.753	0.505	0.342	0.756	0.001
With	0	474.83	0.00	0	969.77	0.00	-	-	-	-	-
**Urbanization level**											
1 (The highest)	18	29, 081.69	61.89	68	73, 454.56	92.57	0.669	0.055	0.010	0.305	<0.001
2	23	30, 277.65	75.96	133	124, 433.19	106.88	0.711	0.141	0.043	0.465	<0.001
3	19	9, 166.57	207.27	13	25, 468.71	51.04	4.061	4.200	0.598	30.598	0.265
4 (The lowest)	0	16, 047.47	0.00	32	37, 086.82	86.28	0.000	0.000	-	-	0.881
**Level of care**											
Hospital center	26	38, 581.75	67.39	103	103, 459.72	99.56	0.677	0.168	0.022	1.298	0.104
Regional hospital	19	29, 565.21	64.26	80	100, 454.25	79.64	0.807	0.544	0.167	1.495	0.899
Local hospital	15	16, 426.42	91.32	63	56, 529.31	111.45	0.819	0.883	0.435	1.802	0.769

Abbreviations: PYs = Person-years; Adjusted HR = Adjusted Hazard ratio: Adjusted for the variables listed in Table 2; CI = confidence interval.

### Comparisons of HRs between estrogen only therapy and combined HRT for the risk of tinnitus

A decreased risk of getting tinnitus, with an incidence rate ratio (IRR) of 0.751, was observed using Cox regressions analysis after adjustments for age and other variables. The individual IRRs of developing tinnitus in the estrogen only therapy group and combined HRT group are respectively 0.822 and 0.654. The adjusted HR (i.e., risk of developing tinnitus) of HRT users (0.505, 95% CI, 0.342–0.756), estrogen only therapy users (0.538, 95% CI, 0.385–0.902) and combined HRT users (0.468, 95% CI, 0.272–0.693) were all significantly lower when compared to HRT nonusers (Table 3).

**Table 3:** Use of HRT and the risk of tinnitus

HRT (With vs. without)	With			Without			Ratio	Adjusted HR	95% CI	95% CI	*P*
Kinds of HRT	Event	PYs	Rate (per 105 PYs)	Event	PYs	Rate (per 105 PYs)					
Total	60	84, 573.38	70.94	246	260, 443.28	94.45	0.751	0.505	0.342	0.756	0.001
Estrogen only	38	48, 965.12	77.61	246	260, 443.28	94.45	0.822	0.538	0.385	0.902	0.003
Estrogen and progestogen	22	35, 608.26	61.78	246	260, 443.28	94.45	0.654	0.468	0.272	0.693	<0.001

Abbreviations: PYs = Person-years; Adjusted HR = Adjusted Hazard ratio: Adjusted for the variables listed in Table 2.; CI = confidence interval.

## DISCUSSION

This is a large-scale retrospective matched-cohort study that explored the association between tinnitus and HRT in menopausal women. Based on this matched-cohort study, we provided evidence that menopausal women under HRT may have a decreased risk of getting tinnitus when compared to those who are not under HRT. The data indicated that HRT may provide potential benefits for the management and prevention of tinnitus after menopause.

The exact mechanism through which HRT reduced the risk of tinnitus in menopausal women remains unclear. Despite the fact that the effects of hormones and HRT have been extensively explored on hearing research, we know very little about the effects of hormones or HRT on tinnitus [7,10–13]. Mechanisms that apply to relevant fields of hearing research indicate that HRT can slow down the decline of hearing through the influence of estrogen [10] and have a protective effect on hearing impairment in menopausal women [12]. However, tinnitus may be affected by more complex pathological changes that take place along the entire auditory pathway. We speculate that potential mechanisms of HRT on tinnitus are the changes in circulating levels of estrogen and progesterone, which modify the chemical composition of the endolymph/perilymph transporting between cochlea chambers, and the regulating electrochemical impulses generated by the hair cells in the cochlea that alters our auditory signals [7, 14].

Nonetheless, there are inconsistent findings in animal [15, 16] and human studies [10, 11, 17] that suggest HRT can negatively affect hearing in aged women [11] and those who are under longer duration of postmenopausal HRT [10]. A case report even indicated that HRT could cause sudden hearing loss [17]. Another study, however, discovered that HRT has neither positive nor negative effect on hearing by detecting auditory brainstem response (ABR) latencies in postmenopausal women [18]. We speculate that the possible negative influence of the hormones on the inner ear may be due to the effect of estrogen on electrolyte balance that disturb inner ear function and also induce a direct effect on the auditory pathways that are suggested to be controlled in part by alterations that take place in neurotransmitter receptor concentrations [17]. Among the very few studies that investigated the relationship between HRT and tinnitus, a study of Korean postmenopausal women provided contradictory findings, indicating that longer durations of HRT are associated with developing tinnitus in postmenopausal women [19]. However, as the study also indicated that individuals with tinnitus were significantly older than those without tinnitus, which is consistent with the fact that HRT can negatively affect hearing in aged women [11] and those who are under longer duration of postmenopausal HRT [10], we cannot truly determine the relationship between HRT and tinnitus without ruling out the age factor. As discrepancies may be due to different databases, ethnic groups and analytic statistics, further experimental and epidemiologic studies will be needed to elucidate the relationship between HRT and tinnitus.

Sleep disturbance is one of the most significant complaints of patients with tinnitus, and evidence shows that insomnia is associated with more distressing tinnitus [20]. An earlier study discovered that the cause of HRT-reduced-tinnitus may be related to the sleep improvement induced by the therapy [7]. As our data recorded no menopausal women with both tinnitus and insomnia, we were unable to assess the relationship between insomnia and tinnitus in menopausal women.

Our data also showed that menopausal women who had higher insured premium (≥35000 NT$) and who lived in higher urbanization region had a higher risk of getting tinnitus. These data also showed demographic developments and that if there is an increase of noise exposure or modern life stress, tinnitus prevalence is expected to continue to increase [6]. In addition, these individuals are more aggressive in seeking medical care with HRT prescriptions and contribute to a significant decreased risk of tinnitus in our survey.

The use of HRT for menopausal syndrome is still controversial and has not been recommended as a standard treatment for tinnitus due to a variety of risks including sudden deafness [7, 17]. HRT has also been reported to associate with a risk of deep vein thrombosis and stroke. Hence, HRT has been avoided or carefully applied among postmenopausal patients with deep vein thrombosis, systemic lupus erythematosus, stroke, severe hypertension, severe liver and kidney dysfunction. This was why less percentage of HRT users are associated with these risks at the end of follow up data (Supplementary Table 1 and Table 1). Our findings imply that otorhinolaryngologists should collaborate closely with gynecologists to monitor menopausal women for HRT applications as that might reduce the risk of tinnitus in a long-term follow up.

Our study still has several shortcomings. First, the dose and duration of HRT were not completely and accurately recorded. Second, the database did not provide the assessment of tinnitus and audiometric results. Third, a population-based study cannot clarify the real mechanism that is associated between HRT and tinnitus in menopausal women. Despite these limitations, this study contributes to the potential benefit of decreased risk of tinnitus in menopausal women with HRT.

## CONCLUSIONS

HRT has been used for control of menopausal symptoms with risk and benefit on specific disorders and symptoms. In this cohort study, we provided alternative evidence that HRT decreased the risk of tinnitus in menopausal women in a long-term and nationwide population-based survey. In conclusion, we believe that HRT may provide a potential benefit on management and prevention of tinnitus on menopausal women.

## MATERIALS AND METHODS

### Study design

This study design is a retrospective matched cohort design.

### Data sources

In this study, we acquired data from the National Health Insurance Research Database (NHIRD, outpatient and hospitalization Longitudinal Health Insurance Database in Taiwan) to investigate the association between HRT and subsequent development of tinnitus in menopausal women over a 10-year period (2000–2010).

### Sample population

The postmenopausal women were selected from 1 January 2000 to 31 December 2010 according to ICD-9-CM codes: 627. The women were diagnosed with tinnitus according to ICD-9-CM code: 388.30. Two study cohorts consisted of randomly selected women who were between 45 and 79 years old during the study period from years 2000 through 2010 and (1) had medical service with a HRT prescription (users) for a menopausal condition (ICD-9: 627) or (2) had medical service without a HRT prescription (nonusers) for a menopausal condition. We excluded all subjects with a diagnosis of tinnitus prior to the enrollment and those who were once prescribed HRT and diagnosed with menopausal syndrome before 2000. In addition, women who had a follow-up duration less than three months were also excluded. We used a propensity-score method to match the selected cohort populations to overcome initial selection bias for HRT users and nonusers (Figure 1).

### HRT medication

HRT users were defined as women having any HRT prescriptions during the study period, and the first HRT prescription date was the index date. A list of all medications containing estrogen only or estrogen combined with progestogens (combined HRT) recommended for HRT and available in Taiwan during the study period was extracted from the database. For HRT nonusers, the index date was the date of the first medical visit during 2000–2010.

### Potential confounding variables

We considered variables believed to affect the risk of tinnitus (age, catastrophic illness, insurance income, otologic diseases, urbanization level and level of medical care) as potential confounders. We also considered comorbidities including otitis media (ICD-9-CM codes 381), Ménière's disease (ICD-9-CM code 386), hearing loss (ICD-9-CM code 389), diabetes mellitus (ICD-9-CM code 250) [DM], hypertension (ICD-9-CM codes 401-405), depression (ICD-9-CM codes 296.2, 296.3, 296.82, 300.4 and 311), insomnia (ICD-9-CM codes 780.52), stroke (ICD-9-CM codes 430-438), dementia (ICD-9-CM codes 290, 294.1 and 331), chronic kidney disease(ICD-9-CM code 585) [CKD], osteoporosis (ICD-9-CM codes 733.00~733.09), nephritis & nephrotic syndrome & nephrosis (ICD-9-CM codes 580-589), hyperlipidemia (ICD-9-CM code 272), systemic lupus erythematosus (ICD-9-CM code 710.0) [SLE], ischemia heart disease (ICD-9-CM codes 410-414) [IHD] and deep vein thrombosis (ICD-9-CM codes 433) [DVT]. These are common risk factors that has been reported in earlier studies [21–23] or observed by the authors in clinical settings.

### Data analysis

All analyses were performed using IBM Statistical Product and Service Solutions (SPSS) for Windows, Version 22.0 (IBM Corp., Armonk, NY, USA). χ^2^ and *t* tests were used to evaluate the distributions of categorical and continuous variables, respectively. Multivariate Cox proportional hazards regression analysis was used to determine the risk of tinnitus, and the results were presented as hazard ratio (HR) with 95% confidence interval (CI). The difference between HRT users and nonusers for the risk of tinnitus was estimated using the Kaplan–Meier method with the log-rank test. A 2-tailed *P* value < 0.05 was considered to indicate statistical significance.

### Ethics statement

The NHIRD encrypts personal patient information to keep privacy and provides researchers with anonymous identification numbers associated with relevant claim information. Patient consent is not required for accessing the NHIRD. The Institutional Review Board of Tri-Service General Hospital approved this study (TSGHIRB No. 2-104-05-126). The committee waived the need for a written informed consent.

## SUPPLEMENTARY MATERIALS TABLE




