# Supplementary material for: Hormone replacement therapy decreases the risk of tinnitus in menopausal women: a nationwide study
Source: Oncotarget. 2018 Feb 8;9(28):19807–16. doi: 10.18632/oncotarget.24452 (PMC5929427; doi:10.18632/oncotarget.24452)
Supplement: Supplementary file 1 [file oncotarget-09-19807-s001.pdf]

Hormone replacement therapy decreases the risk of tinnitus in menopausal women: a nationwide study

SUPPLEMENTARY MATERIALS

Supplementary Table 1: Characteristics of the study participants at the end of follow-up. See Supplementary_Table_1
